# Anti-Inflammatory and Anti-Vascular Leakage Effects by Combination of *Centella asiatica* and *Vitis vinifera* L. Leaf Extracts

**DOI:** 10.1155/2021/7381620

**Published:** 2021-04-14

**Authors:** Myung-Gi Seo, Min-Jeong Jo, Nam In Hong, Min Jung Kim, Kyu Suk Shim, Eunju Shin, Jeong Jun Lee, Sang-Joon Park

**Affiliations:** ^1^Department of Histology, College of Veterinary Medicine, Kyungpook National University, Daegu 41566, Republic of Korea; ^2^Univera Co., Ltd., Econet Center, 78, Achasan-ro, Seongdong-gu, Seoul 04789, Republic of Korea; ^3^Naturetech Co., 450-86, Maebong-Ro, Dongnam-Gu, Cheonan-Si, Chungnam 330-863, Republic of Korea

## Abstract

Venous insufficiency results from several factors responsible for the progression of inflammation and oxidative damage of veins. Recently, natural extracts have been proposed for the treatment of venous insufficiency, but their efficacies have not been fully elucidated. In the present study, we evaluate the combinatorial effects on anti-inflammatory and anti-vascular leakage potential of mixed compositions containing different proportions of *Centella asiatica* extract (CE) and *Vitis vinifera* L. leaf extract (VVE) using an inflammation model of lipopolysaccharide- (LPS-) stimulated RAW264.7 cells and various vascular permeability models in mice (acetic-acid-induced peritoneal vascular model, mustard-oil-stimulated ear vascular model, and carrageenan-induced paw edema model). Pretreatment of CE and VVE in a 1 : 3 combination dose dependently inhibited the production of nitric oxide (NO) and prostaglandin E_2_ (PGE_2_) and mRNA expression of inducible nitric oxide synthase (iNOS) and cyclooxygenase-2 (COX-2) through downregulation of the nuclear factor-*κ*B (NF-*κ*B) pathway in LPS-stimulated RAW264.7 macrophages. In vascular permeability-related mouse models, pretreatment with the CE-VVE 1 : 3 combination significantly reduced the permeability of peritoneal or ear veins caused by acetic acid and mustard oil, respectively. Furthermore, pretreatment of the CE-VVE 1 : 3 combination ameliorated inflammation and edema of the hind paw caused by carrageenan injection. Thus, the combination of CE and VVE showed significant anti-inflammatory qualities and anti-vascular leakage effects. These findings indicate that an optimal combination of CE and VVE may have a more synergistic effect than that of CE or VVE alone as a putative agent against vascular incompetence.

## 1. Introduction

Sustained venous insufficiency causes pathological vascular formations such as spider veins, varicose veins, and venous ulceration as a result of abnormal microcirculation of the lower limbs [1]. These clinical signs are associated with both superficial and deep venous dysfunction. The prevalence of venous insufficiency is mainly seen in the elderly or people in standing occupational groups, and it affects men and women equally. Cases of chronic venous insufficiency cause great economic burden to the society in terms of diagnosis, treatment, reduced working time, and poor quality of life. Although the etiology of venous insufficiency remains unclear, it results from the retention or reflux of blood flow in the veins caused by damage of the venous valves and wall. The mechanism of venous insufficiency formation is complex, and it is generally assumed that oxidative stress and inflammation are the main factors responsible for the changes induced in the lower limb veins. Ongoing exposure to oxidative stress leads to the dysfunction of the vascular endothelium and vascular smooth muscle. Subsequently, vascular inflammation caused by abnormal blood flow or oxidative mediators exacerbates vascular damage.

Despite our limited information of the pathological condition of venous insufficiency, compression therapy and surgical procedure have generally been suggested as treatments for chronic venous insufficiency. Besides these, the pharmacologic treatment of venous insufficiency has been used to treat symptoms such as pain, edema, and vascular inflammation evoked by chronic venous hypertension. Among the pharmacologic agents, herbal medicine has been suggested as a good alternative for treating venous insufficiency due to its wide therapeutic actions.

*Centella asiatica* (L.) Urban is an important herbal medicine that has been widely used for many years in the treatment of cardiovascular diseases [2]. It is anticipated that major constituents of *C. asiatica* may be involved in therapeutic actions against cardiovascular diseases. These terpenoids include asiaticoside, centelloside, madecassoside, asiatic acid, centellic acid, and madecassic acid [3]. In particular, asiaticoside increases wound healing activity through the collagen synthesis in fibroblasts of the vascular walls, which improves their flexibility and blood vessel tension [4]. It has also demonstrated an anti-inflammatory activity and improvement of capillary permeability. Asiaticoside leads to an increase in antioxidant levels, and it participates in the wound healing process [5]. It was shown that the extract from *C. asiatica* influences strengthening the weakened veins in the maintenance of connective tissue. *C. asiatica* was effective against hypertensive microangiopathy and venous insufficiency in addition to decreasing the capillary filtration rate by improving microcirculatory parameters [6]. It, furthermore, has displayed a protective effect on the vascular endothelium and is characterized by antiplatelet activity, which can reduce the negative effects of venous stasis. It also reduces excessive permeability in blood vessels. Improvements in these microcirculatory parameters are usually associated with improved signs and symptoms of venous insufficiency [7].

*Vitis vinifera* L., the grapevine, is the most cultivated plant worldwide and represents an economically important fruit crop for both human consumption and herbal medicine. Traditional use of dried grapes was described for the treatment of cardiovascular conditions such as capillary bleeding, edema, and inflammation [8]. *V. vinifera* L. contains many anthocyanins and proanthocyanidins, sugars, sterols, amino acids, and minerals. Among its bioactive compounds, polyphenols are reported to have anti-inflammatory, antioxidant, and cardioprotective effects [9]. It was recently discovered that *V. vinifera* L. leaves contain a variety of phytoconstituents harboring high antioxidant activity including condensed tannins, phenols, and anthocyanins. The leaves of this plant have anti-inflammatory, antibacterial, and vasorelaxant biological activities [10, 11]. They also have shown efficacy in the treatment of varicose veins at the clinical level [12].

Traditional herbal medicines have been used for the treatment of diseases for thousands of years owing to the various pharmacological activities of complex herbal formulations. Combinatorial effects of herbal medicine seem to derive from the complex interactions between multiple bioactive components within the herbs and/or herbal formulations. Indeed, various pharmacological effects of natural products are synergistically increased by appropriately mixed formulations [13]. However, evidence to support these combinatorial effects remains weak and controversial due to the complexities and misconceptions concerning the synergistic effects of traditional herbal medicine.

Based on this information, we hypothesized that an appropriately mixed formulation of *C. asiatica* extract (CE) and *V. vinifera* L. extract (VVE) would show favorable, combinatorial abilities to suppress inflammation and vascular leakage. Thus, our study evaluated the combinatorial efficacy of a mixture of CE and VVE to induce optimal anti-inflammatory and anti-vascular leakage effects using *in vitro* (LPS-stimulated RAW264.7 cells) and *in vivo* (acetic acid-induced peritoneal or mustard oil-induced vascular permeability and carrageenan-induced hind paw edema) models.

## 2. Materials and Methods

### 2.1. Preparation of Plant Extracts

*C. asiatica* and *V. vinifera* L. leaves used in these experiments were purchased from a herbal market (Danyang, China) and were authenticated by the Korea Institute of Oriental Medicine. A voucher specimen was deposited at the herbarium at the faculty. The method of extraction was as follows: *V. vinifera* L. (200 g) was extracted twice with 40% ethanol (with a 4 h reflux). The extract was filtered through filter paper, concentrated under reduced pressure, and vacuum-dried. *C. asiatica* (200 g) was extracted thrice with 70% ethanol (with a 3-hour 70°C reflux). The extract was also filtered through filter paper, concentrated under reduced pressure, and dried. Combined *V. vinifera* L. and C*. asiatica* extract (VCEC) was prepared by blending dried powders of *C. asiatica* extract (CE) and V. vinifera L. extract (VVE) at a ratio of 1 : 3. The VCEC mixture was analyzed by HPLC using a Phenomenex® Luna C18 column (250 × 4.6 mm, 5 *μ*m). Acetonitril and 1% formic acid were used for analysis of quercetin-3-O-*β*-D-glucuronide (quercetin 3-glucuronide), and acetonitril and 0.3% phosphoric acid were used for analysis of asiaticoside.

### 2.2. Chemicals and Reagents

RAW264.7 mouse monocyte/macrophage cells (No. TIB-71) were purchased from the American Type Culture Collection (ATCC; Rockville, MD, USA), and the DMEM medium and fetal bovine serum (FBS) were purchased from Gibco (a subsidy of Thermo Fisher Scientific, Waltham, MA, USA). Dimethylsulfoxide (DMSO), lipopolysaccharide (LPS, *Escherichia coli* 015B), Evans blue, acetic acid, dexamethasone (DEXA), and *γ*-carrageenan (Carr) were obtained from Sigma-Aldrich (Saint Louis, MO, USA). The Cell Counting Kit-8 (CCK-8) was obtained from Dojindo Molecular Technologies (Rockville, MD, USA). NF-*κ*B p65 (#8242), phospho-I*κ*B-*α* (#2859), I*κ*B-*α* (#4814), Lamin A/C (#2032), and *β*-actin (#4967) antibodies were obtained from Cell Signaling Technology (Danvers, MA, USA). iNOS antibody (NB300-605) was obtained from Novus Biologicals (Littleton, CO, USA). COX-2 antibody (ab15191) was obtained from Abcam (Cambridge, MA, USA). HRP-conjugated goat anti-rabbit and goat-mouse secondary antibodies were obtained from EMD Millipore Corporation (Billerica, MA, USA).

### 2.3. Cell Culture

RAW264.7 cells were cultured in the DMEM medium supplemented with 10% heat-inactivated FBS, glutamine, and antibiotics (penicillin and streptomycin). Cells were maintained at 37°C under 5% CO_2_.

### 2.4. Cell Viability Assay

Cell viability was assessed using a Cell Counting Kit-8 (CCK-8, Dojindo, Japan). Briefly, 96-well plates containing 1 × 10^6^ cells/mL were treated with CE, VVE, or CE-VVE mixture (0.25–400 *µ*g/mL) and incubated for 24 h. CCK-8 was then added to each well, and the plate was again incubated at 37°C for 1 h. The absorbance of each well was recorded at 450 nm using a microplate reader (BioTek, Winooski, VT, USA).

### 2.5. Determination of Nitric Oxide and Prostaglandin E_2_

RAW264.7 cells were seeded at a density of 1 × 10^6^ cells/mL in 96-well plates and incubated for 24 h at 37°C under 5% CO_2_. The medium was then replaced with a serum-free medium. Different concentrations of CE, VVE, or CE-VVE (0.5–400 *µ*g/mL) were prepared in serum-free DMEM to give a total volume of 200 *μ*L in each well of a microtiter plate. After 1 h treatment, cells were stimulated with 500 ng/mL of LPS for 24 h. The presence of nitrite was determined in a cell culture medium using a commercial nitric oxide (NO) detection kit (Promega, Madison, WI, USA). Briefly, 50 *μ*L of cell-free supernatant with an equal volume of Griess reagent in a 96-well plate was incubated at room temperature for 10 min. The absorbance was then measured at 540 nm in a microplate reader (BioTek, Winooski, VT, USA). The amount of nitrite in the media was calculated from a sodium nitrite standard curve. The level of *prostaglandin* E_2_ (PGE_2_) was measured with the manufacturer's protocol of a Prostaglandin E_2_ ELISA Kit (Cayman Chemical, Ann Arbor, MI, USA).

### 2.6. Reverse-Transcriptase Polymerase Chain Reaction (RT-PCR)

RAW264.7 cells were seeded into a 6-well plate (3 mL/plate, 1 × 10^6^ cells/mL) and incubated for 24 h. The following day, cells were pretreated with 200 *µ*g/mL of CE or VVE extracts or CE-VVE combinations (3 : 1, 2 : 1, 1 : 1, 1 : 2, and 1 : 3) for 3 h and then stimulated with 500 ng/mL of LPS for 9 h, respectively. Total RNA from RAW264.7 cells was extracted using the RNA-Bee (Tel-Test, Inc., Friendswood, TX, USA) according to the RNA-Bee manufacturer's protocol. RNA was stored at −70°C until use. A total of 1 *µ*g RNA per sample was reverse transcribed with Moloney murine leukemia virus (M-MuLV) reverse transcriptase (Promega Corporation, Madison, WI, USA), oligo dT-18 primer, dNTP (0.5 *µ*M), and 1U RNase inhibitor. After this reaction cocktail was incubated at 70°C for 5 min, 25°C for 5 min, and 37°C for 60 min in series, M-MuLV reverse transcriptase was inactivated by heating at 70°C for 10 min. Polymerase chain reaction (PCR) was performed in a 2x premixed PCR kit (Bioneer, Seoul, Korea). PCR was performed in a DNA thermo cycler (Perkin Elmer, Foster City, CA, USA) with amplification in 30 cycles of 94°C for 45 sec (denaturing), 56–62°C for 45 sec (annealing), and 72°C for 1 min (primer extension). Primer sequences used are as follows: interleukin (IL)-1*β*: forward, 5′-CTCACAAGCAGAGCACAAGC-3′, reverse, 5′-CTCAGTGCAGGCTATGACCA-3′; IL-6: forward, 5′-TTGCCTTCTTGGGACTGATG-3′, reverse, 5′-CAGAATTGCCATTGCACAACT-3′; iNOS: forward, 5′-CCGGCAAACCCAAGGTCTAC-3′, reverse, 5′-GCATTTCGCTGTCTCCCCAA-3′; COX-2: forward, 5′-GCCAGCAAAGCCTAGAGCAA-3′, reverse, 5′-CCGGCAAACCCAAGGTCTAC-3′; and *β*-actin: forward, 5′-GGGCATTGTGATGGACTCCG-3′, reverse, 5′-TGAGGCCAAGATGGAGCCAC-3′. PCR products were electrophoresed in 1.8% agarose gels and stained with ethidium bromide.

### 2.7. Western Blot Analysis

RAW264.7 cells were cultured in 60 mm dishes for 18 h and incubated with a CE-VVE mixture (400 *µ*g/mL) in the presence or absence of LPS (500 ng/mL) according to the expression time of target proteins. Proteins were separated on polyacrylamide minigels and transferred to nitrocellulose membranes (GE Healthcare Life Sciences, UK) and treated with 5% skim milk in tris-buffered saline with Tween 20 (TBST) for 1 h at room temperature with a rocking plate. Afterward, the membranes were incubated overnight at 4°C with primary antibodies (NF-*κ*B p65, phospho-I*κ*B-*α*, I*κ*B-*α*, Lamin A/C, and *β*-actin) at 1 : 1,000 dilutions with 3% Bovine Serum Albumin (BSA). The membranes were washed three times with TBST for 10 mins and were incubated with species-specific HRP-conjugated secondary antibodies at 1 : 1,000 dilutions in TBST with 5% skim milk for 1 h at room temperature. After washing three times with TBST for 10 mins, the membranes were incubated with an ECL mixture (Pierce Biotechnology, Rockford, IL, USA) and the protein of interest was visualized with the C-DiGit Blot Scanner (Li-COR, Corp. Lincoln, NE, USA).

### 2.8. Histopathological Evaluation

Paw tissues were fixed for 2 days in 10% neutral-buffered formalin at room temperature and then were paraffin-embedded. The tissues were sectioned at a thickness of 3 *µ*m for histology. The sections were deparaffinized with xylene and stained with hematoxylin and eosin (H&E) stain. All stained tissue slides were observed using a Nikon ECLIPS 80i microscope to identify the tissue damage.

### 2.9. Immunohistochemistry

The sections were deparaffinized, and 3% hydrogen peroxide (H_2_O_2_)-methanol was used to block endogenous peroxidase at room temperature for 10 min. Heat-induced antigen retrieval was followed by incubation with a Target Retrieval Solution (Dako, Agilent Technologies, Hamburg, Germany). Cooled slides were washed with phosphate-buffered saline (PBS, pH 7.2) and blocked with normal goat serum (goat origin, included in the Vectastain Elite ABC kit) for 30 min. The sections were then incubated in anti-iNOS (1 : 200) or COX-2 (1 : 200) antibody at 4°C overnight. The sections were washed with PBS (pH 7.2), bound antibodies were detected with the Vectastain Elite ABC kit (Vector Laboratories Inc., Burlingame, CA, USA), and immunoreactive products were visualized with a DAB substrate (Vector Laboratories Inc., Burlingame, CA, USA). Slides were counterstained with hematoxyline (Sigma-Aldrich, St. Louis, MO, USA) and finally mounted using a mounting medium (Sigma-Aldrich, St. Louis, MO, USA). All immunostained tissue slides were observed using a Nikon ECLIPS 80i microscope to identify the tissue damage.

### 2.10. Animals

Seven-week-old male C57BL/6 mice weighing 20 g each were housed in individually ventilated cages, maintained at 22°C–24°C and 40%–50% humidity under a 12 h light/dark cycle. Water and normal diet were provided *ad libitum*. The animals were acclimated to this environment for seven days before experiments began. In each experiment, eight mice per group were utilized throughout the study. All animal experiments were performed in accordance with the National Institutes of Health guidelines for the care and use of laboratory animals and approved by the Kyungpook National University Institutional Animal Care and Use Committee (authorization no. KNU-2017-0083).

### 2.11. Acetic Acid Induced Peritoneal Vascular Permeability in Mice

The acetic acid-induced peritoneal vascular permeability test with moderate modifications was performed as previously reported [14]. Mice were treated orally with CE, VVE, or CE-VVE mixture (400 mg/kg) with different ratios (3 : 1, 1 : 1, or 1 : 3) or injected intraperitoneally with DEXA (2 mg/kg) daily for seven days. The untreated control received only sterile water. Briefly, 1 h after final treatment of CE, VVE, CE-VVE mixtures, or DEXA, each mouse was intravenously injected with 2% Evans blue solution at 0.1 mL/10 g body weight followed by an intraperitoneal injection of 0.78% acetic acid at 0.1 mL/10 g body weight. Thirty min after acetic acid injection, mice were euthanized, and the peritoneal cavity was washed twice each with 5 mL of saline to collect dye leakage in a test tube. Saline washes were filtered and centrifuged for 10 min at 2,000 rpm. Supernatants were collected and measured at 610 nm by using a microplate reader (BioTek). The optical density of the supernatant was measured, and the amount of Evans blue leakage was converted into micrograms.

### 2.12. Mustard Oil-Induced Ear Vascular Permeability in Mice

Mice were treated orally with 400 mg/kg of CE, VVE, or CE-VVE mixture (400 mg/kg) with different ratios (3 : 1, 1 : 1, or 1 : 3) or injected intraperitoneally with DEXA (2 mg/kg) daily for seven days. Simultaneously, mice in the control group received sterile water. Briefly, 1 h after final treatment of CE, VVE, CE-VVE mixtures, or DEXA, each mouse was given 2% Evans blue solution at 0.1 mL/10 g body weight via tail vein injection. Mustard oil was diluted in mineral oil to a concentration of 20% (v/v), and 20 *μ*L was slowly applied topically to the dorsal and ventral surface of the ear with a micropipette as described in previous studies [15]. After 30 min, mice were euthanized, and the center of each ear was evenly punched to form a round, 6 mm-diameter disc. Discs were incubated for 1 day at 60°C in a dry oven, and then, Evans blue dye was extracted in 1 mL of formamide for 48 h. The optical density of the supernatant was measured, and the amount of Evans blue leakage was converted into micrograms.

### 2.13. Carrageenan-Induced Paw Edema in Mice

Paw edema was produced as described previously [16]. Mice received CE, VVE, and CE-VVE mixtures (400 mg/kg) daily for four days in different groups using an oral cannula. Simultaneously, mice in a positive control group received 2 mg/kg of DEXA, and the negative control group received sterile water. Briefly, 1 h after final treatment of CE, VVE, CE-VVE mixtures, or DEXA, 50 *μ*L of 1% carrageenan (type IV; Sigma) was injected into the right hind paw of each mouse. Paw volume was measured using a plethysmometer (Panlab, Barcelona, Spain) before and at 1, 3, and 5 h after carrageenan injection.

### 2.14. Determination of the Combination Index (CI)

Dose-effect relationships were analyzed using the median-effect method described in the work of Chou [17]. The combination index (CI) is a widely accepted method to address the combination effects of two compounds; generally, CI < 1, CI = 1, or CI > 1 represent synergistic, additive, or antagonistic effects, respectively. CI was calculated using “CompuSyn” software according to the classic isobologram equation:

(1) $$\begin{array}{c} \text{CI}=\frac{d_{1}}{D_{1}}+\frac{d_{2}}{D_{2}}. \end{array}$$

In equation (1), *D*_1_ and *D*_2_ are the doses of constituent 1 and constituent 2 alone, required to produce a chosen effect level (usually ED_50_), and *d*_1_ and *d*_2_ are the doses of constituents 1 and 2 in combination required to produce the same effect, respectively.

### 2.15. Statistical Analysis

All data presented in this study are expressed as the means ± standard deviation (SD) from three independent experiments. Statistical analysis was performed using one-way analysis of variance (ANOVA) followed by Dunnett's test. Differences with ^*∗*^*p* < 0.05 or ^*∗∗*^*p* < 0.01 were considered statistically significant. Nonlinear regression analysis was used to generate sigmoidal dose-response curves to calculate IC_50_ values. Data were evaluated using GraphPad Prism 8.

## 3. Results

### 3.1. Effects of CE, VVE, and CE-VVE 1 : 3 Combination on RAW264.7 Cell Viability

**Figure 1:**
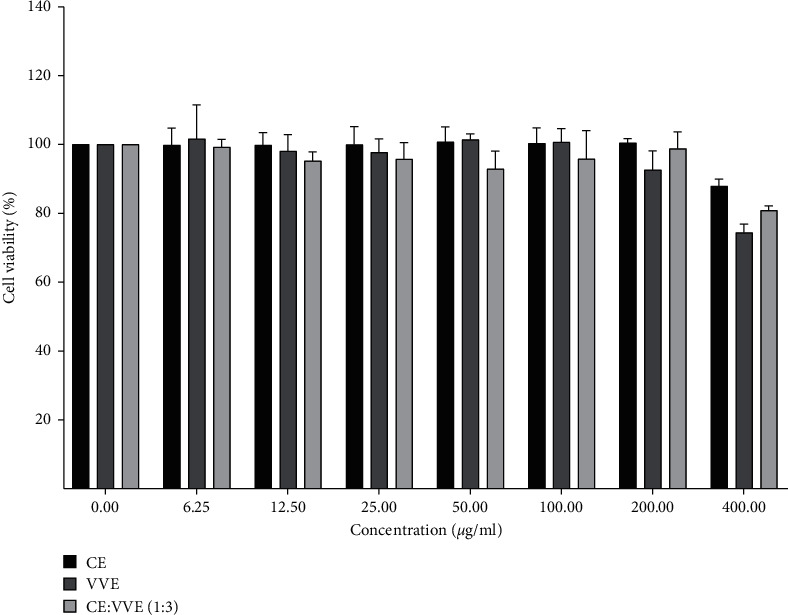
Effects of CE, VVE, or CE-VVE 1 : 3 combination on RAW264.7 cell viability. Cells were plated and cultured for 24 h in the absence or presence of different concentrations of CE, VVE, and CE-VVE 1 : 3 combinations. Each bar represents means ± S.D. of three independent experiments. Abbreviations: CE, *Centella asiatica* extract; VVE, *Vitis vinifera* (L.) extract.

We evaluated the cytotoxicity of CE, VVE, and CE-VVE 1 : 3 combination using CCK-8 assays. Treatment of CE (95.6 ± 0.22 mg/g of Asiaticoside), VVE (11.4 ± 0.16 mg/g of quercetin 3-glucuronide), or CE-VVE 1 : 3 combination (24.9 ± 0.17 mg/g of asiaticoside and 8.5 ± 0.12 mg/g of quercetin 3-glucuronide) did not affect cytotoxicity in RAW264.7 cells at concentrations of ≤400 *µ*g/mL (Figure 1). Based on these results, a concentration range ≤400 *µ*g/mL was chosen for subsequent experiments.

### 3.2. Effect of CE, VVE, and CE-VVE 1 : 3 Combinations on NO and PGE_2_ Production in LPS-Stimulated RAW264.7 Cells

**Figure 2:**
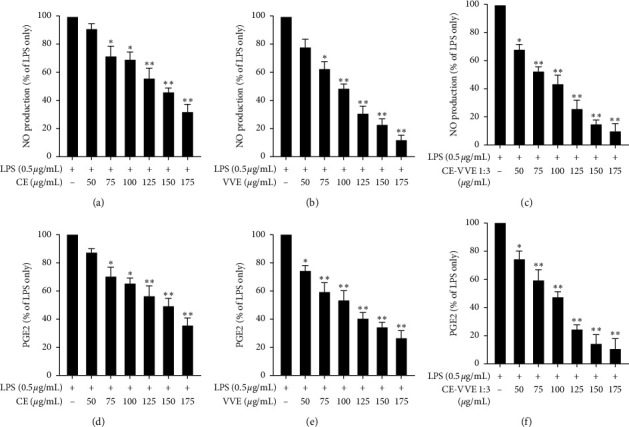
Effects of CE, VVE, or CE-VVE 1 : 3 combination on NO and PGE_2_ production in LPS-stimulated RAW264.7 cells. (a) Cells were plated and cultured for 24 h in the absence or presence of different concentrations of CE, VVE, and CE-VVE 1 : 3 combination. Cells were pretreated with CE, VVE, or CE-VVE 1 : 3 combination (0, 5, 10, 15, 20, 25, and 30 *μ*g/mL) for 1 h and then stimulated with LPS (500 ng/mL) for 24 h. NO production was calculated in culture media by the Griess reagent. (b) PGE_2_ production was quantified by ELISA. All results (*n* = 3) are expressed as the mean ± SD, ^*∗*^*p* < 0.05, ^*∗∗*^*p* < 0.01 vs. LPS-stimulated cells. Abbreviations: CE, *Centella asiatica* extract; LPS, lipopolysaccharide; NO, nitric oxide; PGE_2_, prostaglandin E_2_; VVE, *Vitis vinifera* (L.) extract.

**Figure 3:**
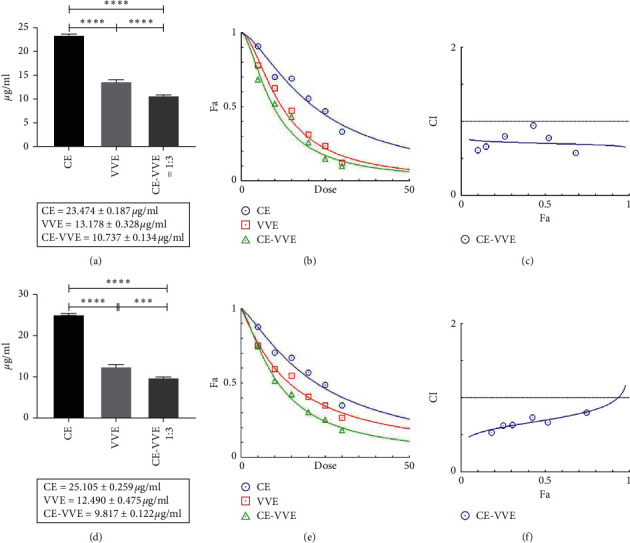
The inhibition effects of CE, VVE, or CE-VVE 1 : 3 on NO and PGE_2_ production in LPS-stimulated RAW264.7 cells. (a) IC50 comparison for NO inhibition among CE, VVE, and CE-VVE 1 : 3 combinations. (b) Dose-effect curves for NO inhibition of CE, VVE, and CE-VVE 1 : 3 combinations. (c) Combination index (CI) values were plotted as a function of fractional inhibition of NO production (fa) by “CompuSyn” software. (d) IC50 comparison for PGE_2_ inhibition among CE, VVE, and CE-VVE 1 : 3 combinations. (e) Dose-effect curves for PGE_2_ inhibition of CE, VVE, and CE-VVE 1 : 3 combinations. (f) Combination index (CI) values were plotted as a function of fractional inhibition of PGE_2_ production (fa) by “CompuSyn” software. Data are expressed as mean ± SD from three individual experiments. ^*∗∗∗*^*p* < 0.001 and ^*∗∗∗∗*^*p* < 0.0001 as compared to each group by one-way ANOVA with multiple comparisons.

To compare the anti-inflammatory activities of CE, VVE, and CE-VVE 1 : 3 combinations, we evaluated NO and PGE_2_ production in LPS-stimulated RAW264.7 cells. CE, VVE, and CE-VVE 1 : 3 combinations all strongly inhibited NO production dose dependently at concentrations between 5 and 30 *µ*g/mL (Figures 2(a)–2(c)). CE and VVE significantly inhibited LPS-stimulated NO production in RAW264.7 cells at concentrations above 10 *µ*g/mL. The IC50 values for CE and VVE in inhibiting NO were 23.47 and 13.18 *µ*g/mL, respectively (Table 1). All CE-VVE combinations with different ratios showed NO inhibitory effects in a dose-dependent manner, with IC50 values ranging from 21.12 to 10.74 *µ*g/mL (Table 1). The IC50 values for all CE-VVE combinations were lower than those of CE and higher than those of VVE except CE-VVE 1 : 3 combination (Table 1, Figures 2(a)–2(c)). Therefore, CE-VVE 1 : 3 showed the strongest NO inhibitory effect among combinations of different ratios, with the lowest IC50 value of 10.74 *µ*g/mL. CompuSyn analysis to determine the synergy, additivity, and antagonism among CE, VVE, and CE-VVE 1 : 3 combinations for NO production is given in Figures 3(a)–3(c). IC50 comparison (a), dose-effect (b), and CI//Fa plot (c) indicate the synergy between the CE and VVE plot shows all the points falling below 1. The combination index (CI) at EC50, EC75, EC90, and EC95 is 0.707, 0.691, 0.676, and 0.666, respectively, indicating the synergy between CE and VVE at all levels. Similar to NO inhibitory data, CE, VVE, and CE-VVE 1 : 3 combinations all significantly suppressed LPS-stimulated PGE_2_ production in a dose-dependent manner at concentrations above 10 *µ*g/mL. The IC50 values for CE and VVE in suppressing PGE_2_ production were 25.11 and 12.49 *µ*g/mL, respectively (Table 1). The IC50 values for all CE-VVE combinations were lower than those of CE and higher than those of VVE except CE-VVE 1 : 3 (Table 1, Figures 3(d)–3(f)). Inhibition of PGE_2_ production was similar to that of NO reduction in this model, implying that the CE-VVE 1 : 3 combination may produce a better anti-inflammatory activity than treatment with CE or VVE alone. CompuSyn analysis to determine the synergy/additivity/antagonism among CE, VVE, and CE-VVE 1 : 3 combinations for PGE_2_ production is given in Figures 3(a)–3(c). IC50 comparison (a), dose-effect (b), and CI//Fa plot (c) indicate the synergy between CE and VVE plot shows all the points falling below 1. The combination index (CI) at EC50, EC75, EC90, and EC95 is 0.701, 0.813, 0.946, and 1.049, respectively, indicating the synergy between CE and VVE.

### 3.3. Effect of CE, VVE, or CE-VVE Combination on the mRNA Expression of Proinflammatory Cytokines, iNOS, and PGE_2_ in LPS-Stimulated RAW264.7 Cells

**Figure 4:**
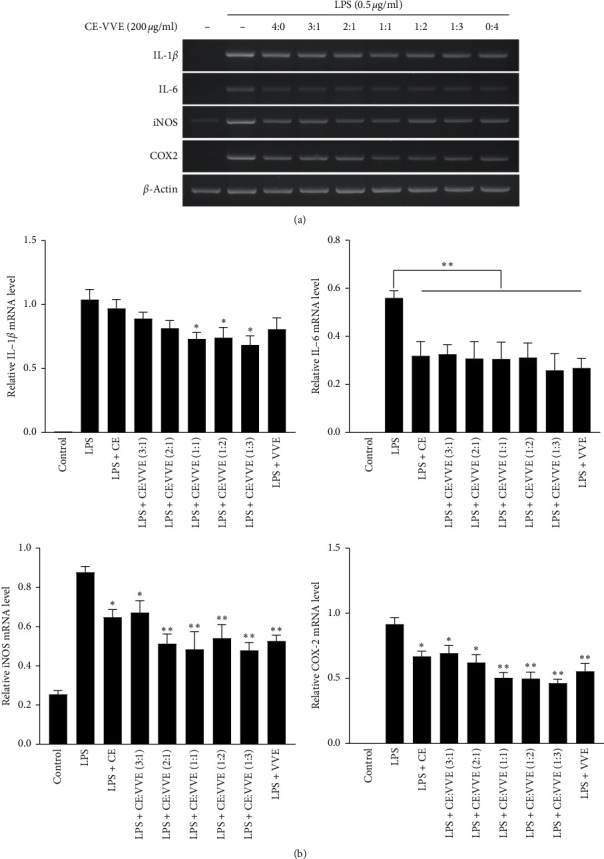
Effect of CE, VVE, or CE-VVE combination on the mRNA expression of proinflammatory cytokines in LPS-stimulated RAW264.7 cells. (a) Cells were cultured in six-well plates and were pretreated with CE, VVE, or CE-VVE combination (200 mg/mL) for 3 h prior to LPS (500 ng/mL) treatment for 9 h. Total RNA was isolated, and mRNA expression levels of IL-1*β*, IL-6, iNOS, and COX-2 were analyzed by RT-PCR. (b) All results (*n* = 3) are expressed as the mean ± SD, ^*∗*^*p* < 0.05, ^*∗∗*^*p* < 0.01 vs. LPS-stimulated cells. Abbreviations: CE, *Centella asiatica* extract; COX-2, cyclooxygenase-2; IL, interleukin; LPS, lipopolysaccharide; iNOS, inducible nitric oxide synthase; and VVE, *Vitis vinifera* (L.) extract.

To evaluate whether CE, VVE, or CE-VVE combination with different ratios affect the expression of proinflammatory cytokines such as IL-1*β*, IL-6, inducible nitric oxide synthase (iNOS), and COX-2, RAW264.7 cells were stimulated with LPS for 9 h after 1 h pretreatment of each extract group. The high expression of IL-1*β* during LPS treatment was slightly inhibited by treatment of CE and VVE alone (Figures 4(a) and 4(b)), but CE-VVE 1 : 1, 1 : 2, and 1 : 3 combinations showed more significant inhibition than CE and VVE alone (Figures 4(a) and 4(b)). As with IL-1*β*, mRNA expression of IL-6 also gradually decreased as the ratio of VVE within the CE-VVE combination increased (Figures 4(a) and 4(b)). The expression of iNOS and COX-2 is closely related to the production of their respective downstream molecules NO and PGE_2_; therefore, we evaluated whether the inhibition of NO or PGE_2_ observed during pretreatment with CE, VVE, or CE-VVE combination could be attributed to the downregulation of iNOS or COX-2 expression. Indeed, as the ratio of VVE within the CE-VVE combination increased, iNOS and COX-2 mRNA expression significantly decreased in LPS-stimulated RAW264.7 cells (Figures 4(a) and 4(b)). These results indicate that suppression of NO or PGE_2_ production may correlate with the inhibition of iNOS or PGE_2_ expression. Thus, although all CE, VVE, and CE-VVE combinations examined herein strongly inhibit the LPS-stimulated inflammatory response in this model, CE-VVE combination 1 : 1, 1 : 2, and 1 : 3 showed a better anti-inflammatory activity than CE and VVE alone. Among them, CE-VVE combination 1 : 3 showed the strongest inhibition on the mRNA expression of proinflammatory cytokines (IL-1*β* and IL-6), iNOS, and COX-2 in LPS-stimulated RAW264.7 cells.

### 3.4. Effect of CE, VVE, and CE-VVE Combinations on the Activation of the NF-*κ*B Pathway in LPS-Stimulated RAW264.7 Cells

**Figure 5:**
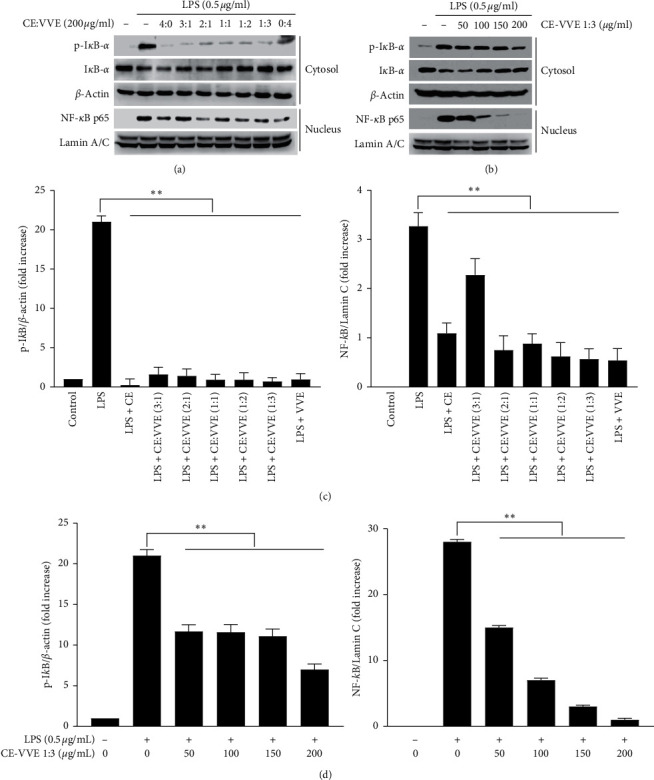
Effect of CE, VVE, or CE-VVE combination on the NF-*κ*B pathway in LPS-stimulated RAW264.7 cells. (a) Cells were cultured in 60 cm dishes and were pretreated with CE, VVE, or CE-VVE combination 1 h prior to LPS (500 ng/mL) treatment for 9 h. Protein expression levels of phosphorylated-I*κ*B or NF-*κ*B were analyzed by western blot using cytosolic and nuclear cell extract. (b) Cells were cultured in 60 cm dishes and were dose-dependently pretreated with a CE-VVE 1 : 3 combination (0, 50, 100, 150, and 200 *μ*g/mL) 1 h prior to LPS (500 ng/mL) treatment for 9 h. Protein expression levels of phosphorylated-I*κ*B or NF-*κ*B were analyzed by western blot using each specific antibody. (c), (d) All results (*n* = 3) are expressed as the mean ± SD, ^*∗*^*p* < 0.05, ^*∗∗*^*p* < 0.01 vs. LPS-stimulated cells. Abbreviations: CE, *Centella asiatica* extract; I*κ*B, nuclear factor of kappa light polypeptide gene enhancer in B-cell inhibitor; LPS, lipopolysaccharide; NF-*κ*B, nuclear factor kappa light chain enhancer of activated B cells; and VVE, *Vitis vinifera* (L.) extract.

Nuclear factor-*κ*B (NF-*κ*B) is a transcriptional factor to regulate proinflammatory gene induction and function in both innate and adaptive immune cells of various inflammatory diseases. Targeting the NF-*κ*B pathway represents an attractive approach for the development of anti-inflammatory drugs. LPS activates this signaling pathway by nuclear translocation of the phosphorylated NF-*κ*B p65 subunit and degradation of the phosphorylated I*κ*B-*α* from NF-*κ*B and I*κ*B-*α* complex in cytosol. Here, we investigated whether CE, VVE, and CE-VVE combinations inhibits the LPS-stimulated degradation of I*κ*B-*α* in RAW264.7 cells by western blotting with specific antibody. Figures 5(a) and 5(c) shows that CE alone, VVE alone, and CE-VVE combinations all strongly reduced LPS-induced I*κ*B-*α* phosphorylation and recovered the I*κ*B-*α* protein level in the cytosol as the ratio of VVE within the CE-VVE combination increased. We also investigated whether CE alone, VVE alone, and CE-VVE combinations inhibit the translocations of the NF-*κ*B p65 subunit from cytosol to the nucleus. It was found that CE, VVE, and CE-VVE combinations significantly block the nuclear translocation of the NF-*κ*B p65 subunit in nuclear extracts by western blotting (Figures 5(a) and 5(c)). Interestingly, the nuclear translocation of the NF-*κ*B p65 subunit was gradually decreased as the ratio of VVE within the CE-VVE combination increased in the same proportional manner of CE-VVE combinations in the inhibition of iNOS, COX-2, IL-1*β*, and IL-6 mRNA expression. Furthermore, to demonstrate dose dependency of CE-VVE combination 1 : 3 and its specificity of the NF-*κ*B pathway, we investigated phosphorylation/degradation of I*κ*B-*α* and nuclear translocation of NF-*κ*B p65 using CE-VVE combination 1 : 3 as a most effective combination. In LPS-stimulated RAW264.7 cells, CE-VVE combination 1 : 3 strongly inhibited phosphorylation of I*κ*B-*α* and nuclear translocation of the NF-*κ*B p65 subunit in a dose-dependent manner (Figures 5(b) and 5(d)).

### 3.5. Effect of CE, VVE, and CE-VVE Combinations on Acetic-Acid-Induced Vascular Permeability

**Figure 6:**
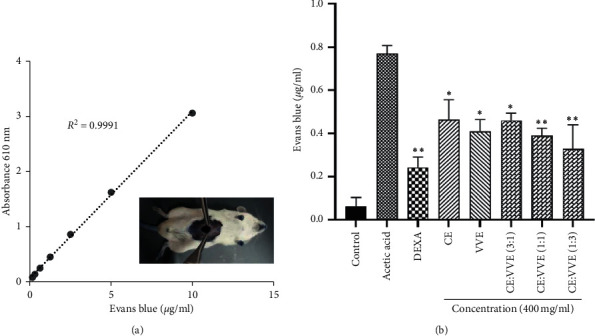
Effect of CE, VVE, or CE-VVE combination on the acetic acid-induced peritoneal vascular permeability in mice. (a) Standard curve for Evans blue solutions of various concentrations (0.156–10 *μ*g/ml). Standard Evans blue solutions were prepared by dissolving dye in 0.9% saline. (b) Vehicle, DEXA, CE, VVE, or CE-VVE combination (400 mg/mL) were administered orally 1 h prior to tail vein injection with 0.1 mL of 2% Evans blue. After 15 min, 0.78% acetic acid was injected into the peritoneal cavity. Quantities of extravasated Evans blue in the mouse peritoneal cavity are plotted on a standard curve for Evans blue as mean ± SD, ^*∗*^*p* < 0.05, ^*∗∗*^*p* < 0.01 vs. acetic-acid-stimulated vehicle group. Abbreviations: CE, *Centella asiatica* extract; VVE, *Vitis vinifera* (L.) extract.

The amount of Evans blue leaked into the peritoneum during acetic acid-induced vascular permeability represented the effect of the extracts against exudation of fluid from blood vessels.  Figure 6(a) depicts the standard curve that was used to determine the concentration of Evans blue in the peritoneum. The Evans blue concentration in the acetic acid-treated group was 0.78 *μ*g/mL. However, the Evans blue concentration in the DEXA-treated group was reduced as a 0.24 *μ*g/mL compared to the acetic acid-treated group. CE alone, VVE alone, and CE-VVE combination (3 : 1, 1 : 1, and 1 : 3) groups showed significantly decreased Evans blue concentrations (by 0.46, 0.41, 0.46, 0.39, and 0.33 *μ*g/mL, respectively) compared to the acetic acid-treated positive control group (*p* < 0.05 or *p* < 0.01; Figure 6(b)). Among CE-VVE combinations, the CE-VVE 1 : 3 combination showed better proportional efficacy to prevent peritoneal leakage.

### 3.6. Effect of CE, VVE, and CE-VVE Combinations on Mustard Oil-Induced Ear Vascular Permeability

**Figure 7:**
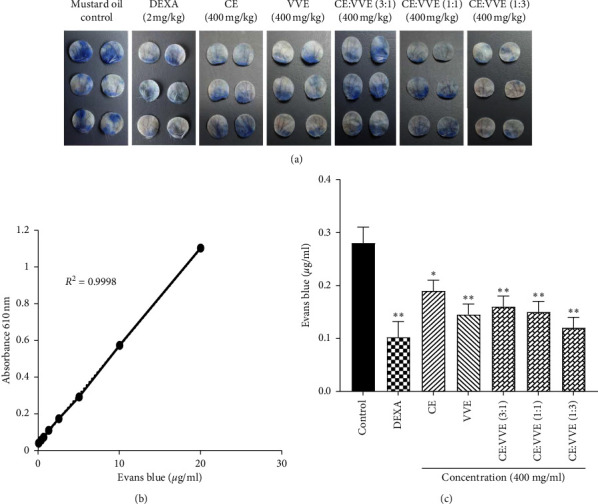
Effect of CE, VVE, or CE-VVE combination on mustard oil-stimulated ear vascular permeability in mice. (a) Representative photographs of punched ear discs from vehicle, DEXA, CE, VVE, or CE-VVE combination (3 : 1, 1 : 1, and 1 : 3) treated groups. Vehicle, CE, VVE, or CE-VVE combination (400 mg/mL) were administered orally 1 h prior to tail vein injection with 0.1 mL of 2% Evans blue. After 30 mins topical application of mustard oil, each ear was evenly punched to form 6 mm-diameter disc. (b) Standard curve for Evans blue solutions of various concentrations (0.156–20 *μ*g/ml). Standard Evans blue solutions were prepared by dissolving dye in formamide. (c) Evenly punched ear discs with 6 mm diameter were dried for 1 day at 60°C in a dry oven, and then, Evans blue dye was extracted in 1 mL of formamide for 48 h The quantities of extravasated Evans blue in mouse ears are plotted on a standard curve for Evans blue as mean ± SD, ^*∗*^*p* < 0.05, ^*∗∗*^*p* < 0.01 vs. mustard oil-stimulated control group. Abbreviations: CE, *Centella asiatica* extract; VVE, *Vitis vinifera* (L.) extract.

Ear vascular permeability was initiated by topical application of 1% mustard oil to both ears of mice. Representative photographs of punched ear discs from vehicle-, DEXA-, CE-, VVE-, or CE-VVE-combination-treated groups show the extravasation of Evans blue dye on the ear veins (Figure 7(a)).  Figure 7(b) depicts the standard curve that was used to determine the concentration of Evans blue leaked from ear vasculature. The Evans blue concentration in the mustard-oil-stimulated group was 0.28 *μ*g/mL. However, leakage of Evans blue concentration in the DEXA group was reduced as a 0.10 *μ*g/mL compared to the mustard-oil-stimulated group. CE alone, VVE alone, or CE-VVE combination (3 : 1, 1 : 1, and 1 : 3) groups showed significantly decreased Evans blue concentrations (by 0.19, 0.14, 0.16, 0.15, and 0.12 *μ*g/mL, respectively) compared to the mustard-oil-treated group (*p* < 0.01; Figure 7(c)). Among CE-VVE combinations, the CE-VVE 1 : 3 combination showed slightly better proportional efficacy in ear vascular permeability.

### 3.7. Effect of CE, VVE, and CE-VVE Combinations in Carrageenan-Induced Hind Paw Edema

**Figure 8:**
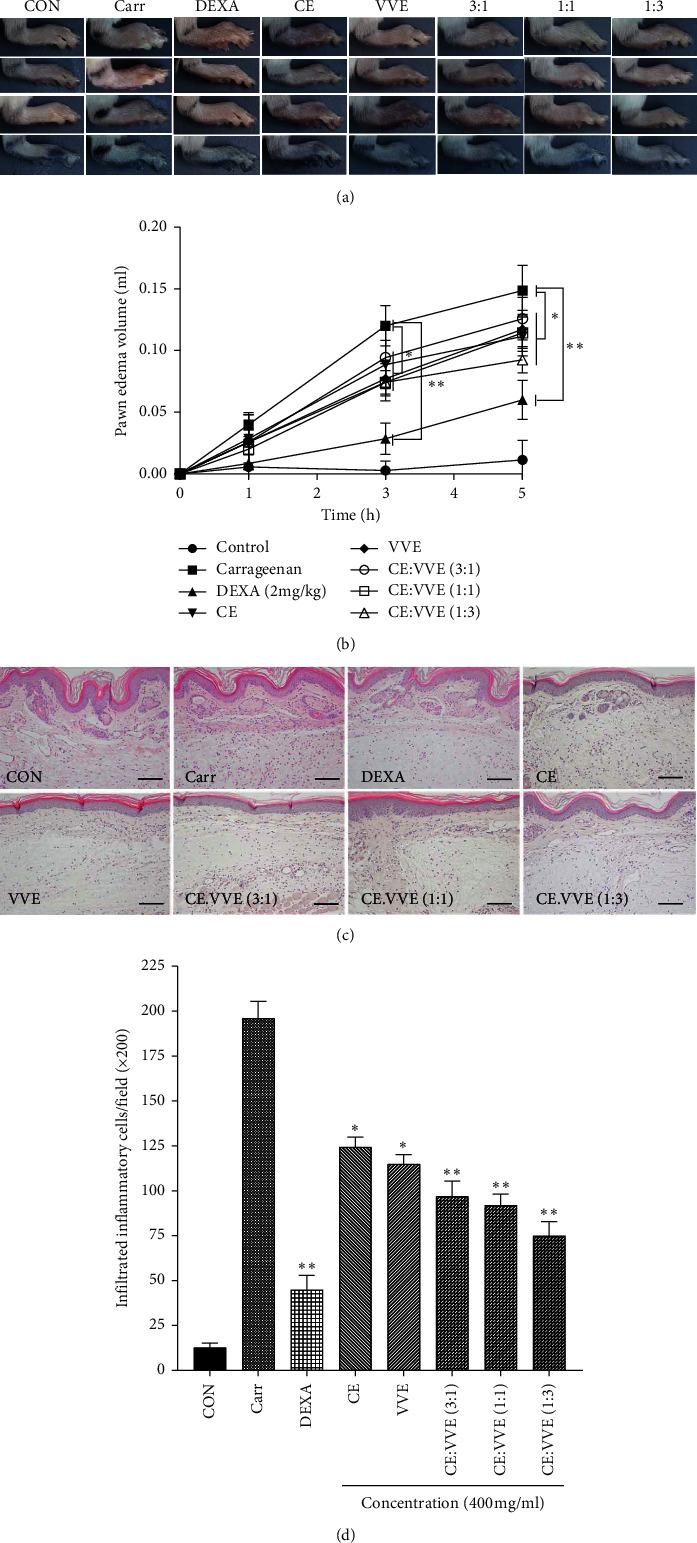
Effect of CE, VVE, or CE-VVE combination on the carrageenan-induced hind paw edema in mice. (a) Photographic images of hind paw of control (saline), carrageenan (saline), DEXA, CE, VVE, or CE-VVE combination (3 : 1, 1 : 1, and 1 : 3) groups at 5 h after carrageenan injection. Footpad edema was induced by 50 *μ*L injection of 1% carrageenan and was evaluated by plethysmometry. CE, VVE, or CE-VVE combination (400 mg/kg) was administered orally 1 h prior to carrageenan injection. Saline and dexamethasone (2 mg/kg) were administered as a negative or positive control. (b) All results were expressed as mean ± SD, ^*∗*^*p* < 0.05, ^*∗∗*^*p* < 0.01 vs. carrageenan group. The increase in paw size was measured 1, 3, and 5 h after carrageenan injection. The time zero corresponds to the time of carrageenan injection. (c) Histopathological findings of mouse hind footpads at 5 h after subcutaneous injection of saline and carrageenan. (d) Number of infiltrated inflammatory cells in the dermis of paw tissues is plotted as mean ± SD, ^*∗*^*p* < 0.05, ^*∗∗*^*p* < 0.01 vs. carr-injected group (original magnification at ×200). Abbreviations: CE, *Centella asiatica* extract; VVE, *Vitis vinifera* (L.) extract.

Carrageenan-induced edema was measured for 5 h at one-hour intervals. The combination of CE-VVE at a 1 : 3 ratio showed significantly better proportional efficacy in carrageenan-induced hind paw edema (*p* < 0.05) than the carrageenan-induced group (Figures 8(a) and 8(b)). The other treatment groups except CE-VVE 1 : 3 tended to decrease but without statistical significance.

### 3.8. Histopathological Evaluation of CE, VVE, and CE-VVE Combinations in Carrageenan-Induced Hind Paw Edema

Histopathological findings were carried out in paw tissue 5 h after the carrageenan-induced edema. The paw tissue revealed marked inflammatory infiltrates, characterized mainly by infiltration of neutrophils and macrophages and accompanied excess edema in the dermis of paw pad (Figure 8(c)). However, our data showed that pretreatment with CE, VVE, and CE-VVE combinations highly decreases inflammatory infiltrates. Among CE, VVE, and CE-VVE combinations, CE-VVE combination 1 : 3 showed lower infiltration of inflammatory cells than other pretreatment groups (Figure 8(d)).

### 3.9. Immunohistochemical Finding of COX-2 and iNOS in Carrageenan-Induced Hind Paw Edema

**Figure 9:**
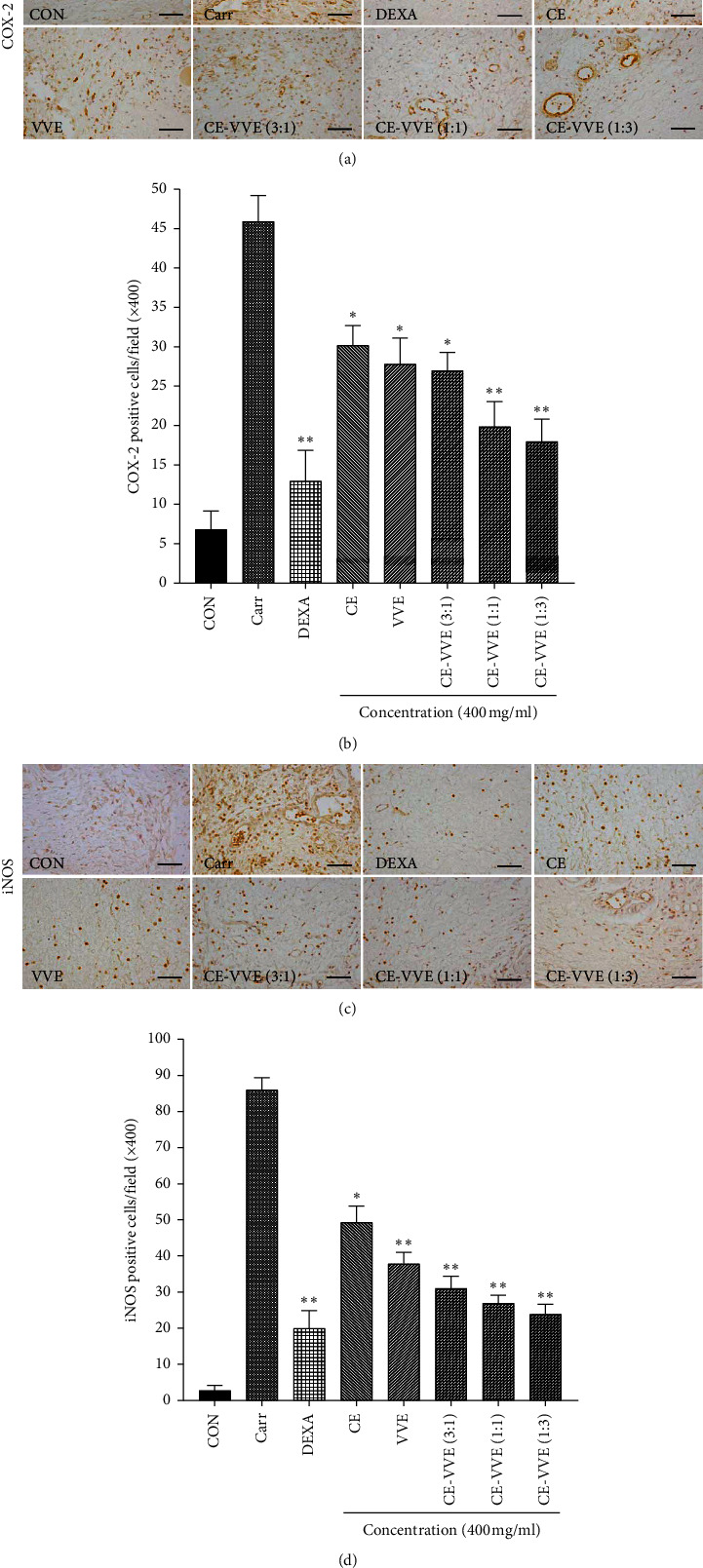
Representative immunohistochemical findings of COX-2 and iNOS on the paw tissues 5 h after carrageenan injection. (a), (b) Marked increases of COX-2 and iNOS positive cells were observed on the dermis of hind footpads in carr-injected mice compared with normal mice. However, these increases of dermal COX-2 and iNOS positive cells were effectively decreased by treatment with dexamethasone and were also dose-dependently affected by treatment with CE, VVE, and CE-VVE combinations. In comparison of extract-treated groups, the CE-VVE 1 : 3 combination shows the best efficacy in paw inflammation. (c), (d) Numbers of COX-2 and iNOS positive cells in the dermis of paw tissues are plotted as mean ± SD, ^*∗*^*p* < 0.05, ^*∗∗*^*p* < 0.01 vs. carr-injected group, respectively (original magnification at ×400).

Immunohistochemical localization of COX-2 and iNOS immunoreactive cells in the dermis of paw skin is shown in Figure 9. COX-2 and iNOS immunoreactive cells were highly increased in the dermis of carrageenan-injected paw skin compared with control paw skin (Figures 9(a) and 9(c)). COX-2 was mainly localized in the infiltrated inflammatory cells, fibroblast-like cells, and endothelial cells of microvessels (Figure 9(a)). Immunopositive cells of iNOS were mainly localized in the infiltrated inflammatory cells and endothelial cells of microvessels (Figure 9(c)). The number of COX-2 and iNOS positive cells in the dermis of paw skin was markedly decreased by treatment of dexamethasone, CE, VVE, and CE-VVE combinations (Figures 9(b) and 9(d)). In comparison of extracts, treatment of the CE-VVE 1 : 3 combination more significantly inhibited iNOS and COX-2 immunopositive cells than that of CE-VVE 3 : 1 and CE-VVE 1 : 1 combinations (Figures 9(b) and 9(d)).

## 4. Discussion

Vascular insufficiency represents progressive pathological changes starting with persistent venous hypertension, oxidative stress, and inflammation; progressing through valve incompetency, venous reflux, edema, and inflammation in the vein wall, ultimately leading to venous hypertensive microangiopathy, varicose vein, and vascular ulceration. Although there is limited understanding in the pathological alteration of venous insufficiency, several therapeutic strategies have been widely used as non-drug-based treatments such as elevation of the extremities, compression therapy, and surgical correction of superficial varicose veins. However, it is assumed that drug-based therapies have the advantage of achieving different treatment strategies depending on the stage of clinical symptoms. Unfortunately, there are no specific drugs with rational therapeutic effects due to the complex mechanisms governing venous insufficiency. Despite the complexity of these mechanisms, oxidative stress and inflammation are accepted as major factors in this disease.

CE and VVE have been used independently as herbal medicines to treat venous diseases such as venous hypertensive microangiopathy, venous hypertension, varicose vein, and chronic venous insufficiency [18–23]. The therapeutic properties of CE are attributed to bioactive components such as triterpenoids (asiaticoside, centelloside, madecassoside, brahmoside, brahminoside, thankuniside, sceffoleoside, centellose, and asiatic acid), sterols, flavonoids, and tannins. Among these components, asiaticoside stimulates the synthesis of collagen and other connective tissue proteins by modulating the action of fibroblasts in the vascular walls, which improves their flexibility and blood vessel tension [3]. In addition, anti-inflammatory actions and the improvement of capillary permeability have been reported. CE has been effective in hypertensive microangiopathy and venous insufficiency as well as in decreasing capillary filtration rates by improving microcirculatory parameters [6]. It also reduces excessive permeability of blood vessels. Improvements in microcirculatory parameters are usually associated with improved signs and symptoms of chronic venous insufficiency [24].

Meanwhile, the pharmacological effect of VVE is attributed to several flavonoids (quercetin-3-O-*β*-glucuronide, quercetin-3-O-*β*-glucoside, and kaempferol-3-glucoside) and phytoconstituents (tannins, phenols, and anthocyanins). Oral administration of VVE improves cutaneous microcirculation and oxygen supply in patients with chronic venous insufficiency [22]. VVE has significantly improved leg edema after 12 weeks, suggesting a multimodal treatment concept for patients with chronic venous insufficiency [12]. Taken together, strong antioxidants and unknown bioactive substances in these extracts are expected to play a role in the application of venous insufficiency.

Recently, a better understanding of the pathological mechanisms underlying venous disease has allowed more rational and efficacious drug development based on oxidative stress and inflammation. Among these, herbal medicine with wide therapeutic actions has shown beneficial effects on vascular abnormality. In addition, more studies are focused on the development of mixed herbal medicine with the aim of increasing the efficacy of plant extracts. It is supposed that the combinatorial effects of herbal medicine stem from the complex interactions between multiple bioactive components within herbal combinations. Various pharmacological effects of natural products have demonstrated synergistic effects through appropriately mixed formulations [13]. However, evidence to support these effects has remained weak and controversial.

In this study, we investigated the anti-inflammatory and antivascular leakage effects of mixed formulas consisting of CE and VVE using both *in vitro* (LPS-stimulated macrophage cells) and *in vivo* (acetic-acid- or mustard-oil-induced vascular permeability and carrageenan-induced hind paw edema) models. We found that various CE and VVE combinations suppressed the secretion of inflammatory mediators such as NO and PGE_2_ in LPS-stimulated RAW264.7 cells. The pharmacological action of various CE and VVE combinations to inhibit NO and PGE_2_ production is believed to be the anti-inflammatory activities of these mixtures. Anti-inflammatory activities for multiple bioactive components from CE and VVE have been elucidated in numerous preclinical studies [25, 26]. In addition, a previous clinical study demonstrates that veins from varicosed patients produce higher amounts of PGE_2_ in comparison with veins from nonvaricosed patients, indicating the importance of the COX pathway in the walls of varicose veins [27].

NF-*κ*B is a major signal transducer in the inflammatory response, and its activation and nuclear translocation upregulate the transcription of a broad range of proinflammatory genes. In this study, NF-*κ*B nuclear translocation was strongly decreased by treatment with CE-VVE combinations (1 : 2 and 1 : 3) or VVE alone in proportion to the amount of VVE within the CE-VVE ratios. This trend corresponds with the expression of iNOS, COX-2, IL-1*β*, and IL-6 mRNA. Different ratios of CE and VVE in combination are expected to regulate the transcription of proinflammatory genes accordingly. Nevertheless, these results indicate that CE and VVE regulate the expression of a broad spectrum of proinflammatory genes through modulation of the NF-*κ*B pathway, one of the representative inflammatory signaling pathways.

The acetic-acid- or mustard-oil-induced vascular permeability test is a general approach to assess vascular leakage by acute vascular damage. Acetic acid or mustard oil can cause an increase in the levels of mediators such as prostaglandins, serotonin, and histamine in peritoneal fluids. This pathophysiological change leads to dilation of capillaries and an increase in vascular permeability within the peritoneal cavity or ear dermis [14]. We found that oral administration of these extracts significantly inhibited the increased capillary permeability induced by acetic acid or mustard oil in mice, suggesting that these extracts may exert an antivascular leakage effect by inhibiting the inflammatory mediators or oxidative stress present in the acute stage. Inhibition of capillary permeability is further enhanced by the combination of CE and VVE rather than single treatment with either CE or VVE. The greatest inhibitory effect was found at a ratio of 1 : 3 (CE : VVE). Although the mechanism of this combinatorial effect of CE and VVE is not clear, this synergistic effect was found to be much greater during the complexity of the *in vivo* models than *in vitro* experiment. These pharmacological effects of natural products may be more enhanced by the application of appropriately mixed formulations owing to the various pharmacological activities of complex herbal formulations.

Carrageenan-induced paw edema is an apt model of acute inflammation and is, therefore, suitable for testing anti-inflammatory and antiedematous activity [28]. Paw edema after subplantar injection of carrageenan progresses as a biphasic reaction by inflammatory mediators or early inflammatory cells [29]. The early phase (1-2 h) occurs from the release of inflammatory mediators such as histamine and serotonin locally at the site of carrageenan injection. In the late phase (4–6 h), edema is caused by an increase of NO and PGE_2_ produced by infiltrating inflammatory cells as well as various parenchymal cells around the damaged tissue. All herbal extracts studied herein significantly reduced paw edema at 3 and 5 h after carrageenan injection. The combination of CE-VVE at 1 : 3 showed the best inhibitory efficacy over the entire experimental period. This result indicates that the bioactive components within combined CE-VVE treatment may enhance the antiedematous activity through inhibition of the release of inflammatory mediators such as NO and PGE_2_ as well as proinflammatory cytokines.

## 5. Conclusions

In conclusion, we found that the combination of CE and VVE synergistically enhanced anti-inflammatory and antivascular leakage effects by reducing inflammatory mediators. Especially, treatment of CE-VVE combination 1 : 3 significantly decreased NO, PGE_2_, and inflammatory mediators (IL-1*β*, IL-6, iNOS, and COX2) in LPS-stimulated RAW264.7 cells, and it also reduced peritoneal and ear vascular permeability induced by acetic acid or mustard oil, respectively. Furthermore, this combination alleviated carrageenan-induced edematous inflammation with CE-VVE-ratio dependency. Therefore, the combination of CE and VVE presents a promising herbal medicine for the prevention of inflammation and abnormal vascular permeability related to venous insufficiency.

## Figures and Tables

**Table 1:** IC50 values of CE-VVE combinations in inhibiting NO and PGE_2_ induced by LPS on RAW264.7 cells.

CE : VVE	IC_50_ (*µ*g/mL)	
	NO	PGE_2_
4 : 0	23.47	25.11
3 : 1	21.12	25.36
2 : 1	18.92	22.54
1 : 1	17.32	17.89
1 : 2	15.78	14.01
1 : 3	10.74	9.82
0 : 4	13.18	12.49

## Data Availability

The data used to support the findings of this study are available from the corresponding author upon request.
